# Desire for birth spacing or limiting and non-use of long acting and permanent contraceptive methods among married women of reproductive age in Aksum Town, North Ethiopia

**DOI:** 10.1186/s40834-016-0033-2

**Published:** 2016-11-15

**Authors:** Kebede Haile, Meresa Gebremedhin, Haileselasie Berhane, Tirhas Gebremedhin, Alem Abraha, Negassie Berhe, Tewodros Haile, Goitom Gigar, Yonas Girma

**Affiliations:** 1 College of Health Science, Aksum University, P.O. Box 1010, Aksum, Ethiopia; 2 College of Health Science, Addis Ababa University, Addis Ababa, Ethiopia; 3 Tigray Regional Health Bureau, Mekelle, Ethiopia; 4 Center of International Reproductive Health Training (CIRHT), Bahir Dar University, Bahir Dar, Ethiopia

**Keywords:** Desire for birth spacing or limiting, Non-use of LAPMs, Aksum, Ethiopia

## Abstract

**Background:**

Long acting and permanent contraceptive methods are the most effective family planning (FP) methods to prevent pregnancy and thereby averting adverse consequences of too many and ill-timed pregnancies. However, long acting and permanent contraceptive methods (LAPMs) are underutilized in Ethiopia for little documented reasons. Therefore, this study is aimed to assess magnitude and factors associated with desire for birth spacing for at least 2 years or limiting child bearing and non-use of LAPMs among married women of reproductive age in Aksum town, Northern Ethiopia.

**Methods:**

A community-based cross-sectional study was conducted in Aksum town, North Ethiopia from May to June, 2015 among 779 randomly selected married women of reproductive age. Data were collected using interviewer administered pre-tested questionnaire. Data were entered using Epi-Info version 6.04 and exported to SPSS version 16 for analysis. Multivariate logistic regression models were fitted to identify factors associated with desire for birth spacing or limiting and not using LAPMs.

**Results:**

The total desire for birth spacing or limiting was 69 % and amongst those women 85.2 % were not using LAPM. Education, occupation, husband’s attitude towards LAPMs, age, number of pregnancy, regular media exposure and decider on the number of children to bear were significantly associated with desire for birth spacing or limiting. Moreover; education, occupation, husband’s attitude towards LAPMs, discussion on family planning with husband, knowledge, attitude and intention to use LAPMs were significantly associated with not using LAPMs.

**Conclusion:**

Desire for birth spacing or limiting and not using LAPMs is very high in the study area. Therefore, increasing access to family planning information and services with special emphasis on LAPMs and male involvement in the program are very important.

## Background

Long-acting and permanent methods of contraception (LAPMs) are modern family planning (FP) methods that prevent pregnancy for three or more years per application. LAPMs include intrauterine contraceptive devices (IUCDs), implants, female sterilization, and male sterilization [1]. LAPMs are safe, cost effective, convenient for users and 3–60 times more effective in preventing pregnancy than short acting methods (pills, injectable hormones and condoms) during a year of typical use [2, 3]. Effective contraceptive could prevent as many as one in every three maternal deaths by allowing women to delay motherhood, space births, avoid unintended pregnancies and abortions, and stop childbearing when they have reached their desired family size [4].

In the developing region, 57 % of the 1.6 billion women of reproductive age desire to space births for at least 2 years or to limit childbearing at all [5]. Satisfying this total demand for contraception could prevent 52 million unintended pregnancies per year which would result in 500,000 fewer new born deaths and 70,000 fewer maternal deaths each year from the current status. However, about 26 % the total women who desire to space or limit births in developing region and 58 % in sub-Saharan Africa lack access to the modern methods of contraception [5, 6]. Even among the users of modern contraception methods, short acting methods of contraception, the less effective methods, are widely used (27 %) [2, 3, 5, 7, 8]. Furthermore, about 20–30 % of women who use short acting methods of contraception stop within 2 years of starting due to health concerns. Many of these women could benefit from switching to LAPMs [9].

In the case of Ethiopia, nearly 75 % of married women of reproductive age desire to space births for at least two years or to limit childbearing at all but not more than 5 % are using LAPMs [10]. Moreover, Ethiopia continues to be one of the most populous countries in the world with a total population of 87.1 million and total fertility rate of 4.8 children per reproductive age group women [11]. According to Ethiopian Demographic and Health Survey (EDHS) report, Ethiopia is one of the Sub-Saharan Africa countries with the highest maternal mortality (676 per 100,000 live births) and neonatal mortality (37 per 1,000 live births) rates [10].

Therefore, to solve the aforementioned health problems in particular and to achieve the development goals in general, it is crucial to promote desire to space or limit births and let all women who desire to space or limit births access to LAPMs. Different factors may contribute to low desire for birth spacing or limiting and high non-use of LAPMs. As indicated by some studies conducted elsewhere; socio- demographic characteristics, reproductive health characteristics, knowledge, attitude, myths and misconceptions may affect desire for birth spacing or limiting and contribute to non-use of LAPMs [12–29].

Given the prevailing low utilization of LAPMs in Ethiopia, analysis of desire for birth spacing or limiting and non-use of LAPMs and a critical assessment of the underlying factors are very important. This information would have an important role in designing effective programs to heighten desire for birth spacing or limiting and reduce the prevailing high non-use of LAPMs. However, little has been documented so far in Ethiopia with regard to the aforementioned issues. Thus, this study is aimed to assess the magnitude and factors associated with desire for birth spacing for at least 2 years or limiting child bearing and non-use of LAPMs among married women of reproductive age in Aksum town, Northern Ethiopia.

## Methods

### Study design and setting

This community based cross sectional study was conducted from May to June, 2015 among married women of reproductive age (15–49 years) in Aksum town. Aksum town is located in the northern part of Ethiopia between 14, 1297 (147’46.920”N) latitude and 38, 7158 (3842’56.880”E) longitude at a distance of 1010 km away from Addis Ababa. According to the 2007 report of Central Statistical Agency of Ethiopia, the total population of Aksum town is 60,766, with 30,991 (51.0 %) females and 29,775 (49.0 %) males. Administratively the town is divided in to four Kebeles (small local administrative units) [30].

### Sample size and sampling procedure

The sample size was determined using a single population proportion formula assuming; 95 % level of confidence, proportion of women who desire to space or limit births of 50 %, a design effect of 2 and non-response rate of 5 %. This gave a final sample size of 806 married women of reproductive age. A two-stage sampling approach was used; where first 2 kebeles were selected randomly from the total 4 kebeles of Aksum town. In the selected kebeles, census was done to identify households where married women of reproductive age live. Then, the total sample size was allocated to each of the randomly selected kebeles based on probability proportional to size allocation. Secondly, using the sampling frame from the census of each respective kebele, 806 women who fulfilled the inclusion criteria were selected by simple random sampling technique. In case of absence of the eligible woman in the selected household, repeated attempts were made to get the woman. If the respondent could not be interviewed after 3 attempts, the woman was considered as non-respondent. When two or more married women were in a selected household, only one of them was considered by lottery method to participate in the study, to avoid intra-class correlation. Eventually, a total of 779 women were participated in this study.

### Measurement

Data were collected using interviewer administered structured questionnaire adapted from different literatures [10, 12–14, 19, 31]. The questionnaire was translated and contextualized to the local situation. The contents of the questionnaire included: socio-demographic factors, sexual and reproductive characteristics, knowledge, attitude and practice of family planning methods. Prior to data collection, the questionnaire was pre-tested on 5 % of the sample on similar population in one of the non-study kebeles. Based on the results of pretest, the time required for interviewing each participant was estimated and the skip pattern of some of the questions was corrected. The reliability of the items on attitude scale of the questionnaire was also tested using Cronbach's alpha after pre-test and a value superior or equal to 0.7 was considered as reliable. The Cronbach’s alpha coefficient of attitude items was 0.75.

The data were collected by eight female diploma nurses and the data collection was entirely supervised by two BSc Nurses. Furthermore, the data collection process was closely monitored by the principal investigators. Both data collectors and supervisors were trained for 2 days on the objectives of the study, sampling technique, data collection tool and techniques of collecting data to maintain precaution throughout the study. To reduce non-response or reporting biases, interviews were conducted either in private rooms or places where other people could not overhear. Daily meetings were also held between the principal investigators and enumerators to troubleshoot problems that arose in the data collection process. In addition, inspection for completeness and quality of data collection was carried out daily by the supervisors and feedback was provided to data collectors.

Married women’s knowledge on LAPMs was assessed by asking 12 questions with ‘yes/no’ answers adapted from different literatures [10, 19, 31]. For each knowledge question a score of 1 was given to correct and 0 to incorrect responses. The final score was computed by summing all correct answers. Finally, to evaluate knowledge of the married women, it was categorized as “high” - for those who knew 70 % and above, “moderate” - for those who knew 40 - 69 % and “low”- for those who knew less than 40 % of the total knowledge questions [31].

Married women’s attitude towards LAPMs was assessed using 10 items rated on a five-point Likert scale adapted from different literatures [19, 31]. Attitude score was computed using the above 10 items whose theoretical value ranges from 10 to 50. This scoring was subsequently reversed for negatively stated statements. Finally, respondents who scored above the median attitude score of the sampled population, which is 31,were labeled as having a positive attitude whereas respondents with an attitude score of less than or equals to the median score were labeled as having a negative attitude [31].

### Data processing and analysis

Data were entered and cleaned using Epi- Info version 6.04, and transferred to SPSS 16 statistical software package for analysis. Descriptive analysis was used to describe the data. The dependent variables were desire for birth spacing or limiting, and LAPMs use. Desire for birth spacing or limiting was coded as 0 for women who have no desire for birth spacing or limiting and 1 for those who have desire for birth spacing or limiting. LAPMs use was coded as 0 for women who have desire for birth spacing or limiting and using LAPMs and 1 for those who have desire for birth spacing or limiting but not using LAPMs. Bivariate analysis was used to see the unadjusted effect of each factor on each of the dependent variables of the study. The independent variables with a *p* ≤ 0.05 in the bivariate analyses with each of the dependent variables were fitted in to a multivariate logistic regression model to identify their independent effect on desire for birth spacing or limiting, and not using LAPMs among women who desire to space or limiting birth. Odds ratio with 95 % confidence interval were calculated both to assess the association and measure the strength of the association between the explanatory and outcome variables.

## Results

### Socio-demographic characteristics of respondents

Out of the total 806 sampled married women, 779 women were included in the study with response rate of 96.7 %. The mean (± SD) age of the respondents was 30.01 (±7.29) years. Majority of the respondents were Orthodox (91.2 %) in religion, house wives (63.2 %) in occupation and at least primary school (77.7 %) in education. The median monthly family income was 1000.0 ETB with a range of 250.0–5000.0 ETB (Table 1).

**Table 1:** Socio-demographic characteristics of married women of reproductive age in Aksum town, North Ethiopia, 2015, (*N* = 779)

Characteristics	Number	Percent
Age		
15-24	198	25.4
25-34	334	42.9
35-44	215	27.6
≥ 45	32	4.1
Religion		
Orthodox	711	91.2
Muslim	66	8.5
Others^a^	2	0.3
Educational status		
No formal education	174	22.3
Primary	280	36.0
Secondary	229	29.4
Above secondary	96	12.3
Occupation		
House wife	492	63.2
Merchant	137	17.6
Employed^b^	97	12.4
Daily laborer	36	4.6
Student	17	2.2
Husband’s educational status		
No formal education	182	23.4
Primary education	261	33.5
Secondary education	176	22.6
Above secondary education	160	20.5
Husband’s occupational status		
Employed^b^	260	33.4
Merchant	202	25.9
Daily laborer	197	25.3
Farmer	41	5.3
Private work	37	4.7
Student	30	3.9
Priest	12	1.5
Monthly family income		
≤ 500 ETB	168	21.6
> 500 ETB	611	78.4
Regular media exposure ^c^		
Yes	674	86.5
No	105	13.5
Visited and given health education on FP by HEWs		
Yes	123	15.8
No	656	84.2

1 USD = 20.95 ETB,^a^ = protestant, catholic, ^b^ = both governmental and nongovernmental, ^c^ = listen to radio and/or watch TV at least once per week

### Reproductive characteristics of respondents

Out of the total respondents, 716 (91.9 %) had ever been pregnant. The median number of living children was 2 with 0 and 10 minimum and maximum alive children, respectively. More than two third, 539 (69.2 %) didn’t desire to have a child within the next 2 years. Pertaining to the fertility related decision making, 592 (76.0 %) reported joint decision making by both husband and wife on the number of children to bear. About 581 (74.6 %) of the respondents reported that they had discussed FP methods with their husband at least once in the past 6 months. Nearly three fourth (72.5 %) of the respondents believed that their husband approves the use of LAPMs. Moreover, majority (70.4 %) reported that they jointly decide with their husband on which type of contraceptive to use (Table 2).

**Table 2:** Reproductive characteristics of married women of reproductive age in Aksum town, North Ethiopia, 2015, (*N* = 779)

Characteristics	Number	Percent
Number of pregnancy		
None	63	8.1
1-2	345	44.3
3-4	233	29.9
≥ 5	138	17.7
Number of abortion (*n* = 87)		
1	55	63.2
≥ 2	32	36.8
Number of living children		
None	67	8.6
1-2	369	47.3
3-4	244	31.3
≥ 5	99	12.8
Desire for more children		
Yes	500	64.2
No	279	35.8
Desired time for having additional child (*n* = 500)		
Within two years	240	48.0
After two years	260	52.0
Decider on when to have another child		
Husband	13	1.7
Wife	142	18.2
Both wife and husband jointly	624	80.1
Decider on number of children to bear		
Husband	29	3.7
Wife	158	20.3
Both wife and husband jointly	592	76.0
Discussed FP methods with husband		
No, never	198	25.4
Yes, once/twice	397	51.0
Yes, more often	184	23.6
Husband’s attitude towards LAPMs use		
Approve	565	72.5
Disapprove	59	7.6
Don’t know	155	19.9
Decider on type of contraceptive to be used		
Husband	40	5.1
Wife	191	24.5
Both wife and husband jointly	548	70.4

### Knowledge and attitude of respondents

Seven hundred forty seven (95.9 %) of the total respondents reported that they had heard of LAPMs, and 578 (74.2 %) of them were familiar with at least one of the LAPMs. The most commonly known LAPMs was implant which account for 535 (71.6 %), followed by IUCD 433 (58.0 %). Major source of information about LAPMs was public health facilities 691 (92.5 %), followed by HEWs 553 (74 %). Generally, based on the composite scores of knowledge and attitude, 501 (64.3 %) of the respondents had moderate or high knowledge on LAPMs whereas half 390 (50.1 %) had positive attitude towards practicing LAPMs (Table 3).

**Table 3:** Knowledge and attitude towards long acting and permanent contraceptive methods among married women of reproductive age in Aksum town, North Ethiopia, 2015

Variables	Responses	Number (Percent)
Ever heard of LAPMs (*n* = 779)	Yes	747 (95.9)
	No	32 (4.1)
^a^Source of information on LAPMs (*n* = 779)	Public health facilities	691 (88.7)
	HEWs	553 (71.0)
	Media	489 (62.8)
	School	122 (15.7)
Know at least one LAPM (*n* = 779)	Yes	578 (74.2)
	No	201 (25.8)
^a^Type of LAPM known (*n* = 779)	Implants	535 (68.7)
	IUCD	433 (55.6)
	Female sterilization	162 (20.8)
Knowledge score (composite)	Low	278 (35.7)
	Moderate	212 (27.2)
	High	289 (37.1)
Attitude score (composite)	Negative	389 (49.9)
	Positive	390 (50.1)

^a^Each of the percentages does not add up to 100.0 because respondents could choose several responses which could be spontaneous or prompted

### Desire for birth spacing or limiting and not using long acting and permanent contraceptive methods

**Fig. 1:**
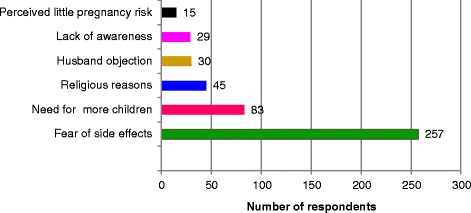
Reasons for not using LAPMs among women of reproductive age who had desire for birth spacing or limiting in Aksum town, North Ethiopia, 2015 (*N* = 539)

The total desire for birth spacing or limiting was 69.2 %; 35.8 % for limiting and 33.4 % for spacing. Of the total women who had desire for birth spacing or limiting, 80 (14.8 %) were using the method whereas the rest 459 (85.2 %) women were not using LAPMs; 48.8 % for spacing and 51.2 % for limiting. The three main reasons cited by the respondents for not using LAPMs were fear of side effect 257 (56.0 %), need for many children 83 (18.1 %) and religious reasons 45 (9.8 %) (Fig. 1).

### Factors associated with desire for birth spacing or limiting and not using long acting and permanent contraceptive methods

Results of multivariate logistic regression analysis showed age, education, occupation; daily labor, regular media exposure, number of lifetime pregnancy, joint decision making on the number of children to bear and husband’s attitude towards LAPMs use were positively associated with desire for birth spacing or limiting. That is, being ≥35 years old (AOR = 2.10, 95 % CI: [1.21, 3.64]), attending primary level of education (AOR = 1.70, 95 % CI: [1.09, 3.56]), being daily laborer in occupation (AOR = 2.83, 95 % CI: [1.02, 7.87]), being regularly exposed to media (AOR = 2.03, 95 % CI: [1.26, 3.26]), having ≥3 lifetime pregnancy (AOR = 1.73, 95 % CI: [1.15, 2.60], joint decision making on the number of children to bear (AOR = 2.43, 95 % CI: [1.58, 3.53]) and unfavorable/unknown husband’s attitude towards LAPMs use (AOR = 9.57, 95 % CI: [4.91, 17.20]) were more likely to desire for birth spacing or limiting compared to their counterparts (Table 4).

**Table 4:** Factors associated with desire for birth spacing or limiting among married women of reproductive age in Aksum town, North Ethiopia, 2015 (*N* = 779)

Variables	Desire for birth spacing or limiting		COR (95 % CI)	AOR (95 % CI)^a^
	Yes (n)	No (n)		
Age				
< 25 25-34 ≥ 35	110 234 195	88 100 52	1 1.87 (1.30, 2.70) 3.00 (1.98, 4.54)	1 1.46 (0.97,2.20) 2.10 (1.21,3.64)**
Education				
No formal education Primary education Secondary education Above secondary education	130 203 150 56	44 77 79 40	2.11 (1.24, 3.59) 1.88 (1.16, 3.05) 1.36 (0.83,2.21) 1	1.97 (0.85,3.43) 1.70 (1.09,3.56)* 1.48 (0.84,2.60) 1
Occupation				
Daily laborer Merchant Employed Student Housewife	31 80 68 8 352	5 57 29 9 140	2.47 (0.94,6.47) 0.56 (0.38,0.83) 0.93 (0.58,1.50) 0.35 (0.13,0.94) 1	2.83 (1.02,7.87)* 0.52 (0.34,0.79)** 1.06 (0.59,1.88) 0.45 (0.16,1.22) 1
Regular media exposure				
Yes No	480 59	194 46	1.93 (1.27,2.94) 1	2.03 (1.26,3.26)** 1
Number of pregnancy				
< 3 ≥ 3	243 296	165 75	1 2.68 (1.94,3.70)	1 1.73 (1.15,2.60)**
Decider on number of children to bear				
Self/husband Both wife and husband jointly	96 443	91 149	1 2.78 (1.98,3.91)	1 2.43 (1.58,3.53)***
Knowledge on LAPMs				
High Moderate Low	214 150 175	75 62 103	1.65 (1.10,2.47) 1.46 (0.99,2.15) 1	1.36 (0.86,2.15) 1.23 (0.79,1.93) 1
Attitude towards LAPMs use				
Positive Negative	284 255	106 134	1.41 (1.04,1.91) 1	1.18 (0.80,1.72) 1
Ever use of modern contraception methods				
Yes No	423 116	161 79	1.79 (1.28,2.51) 1	1.42 (0.97,2.07) 1
Husband’s attitude towards LAPMs use				
Approve Disapprove/don’t know	338 201	227 13	1 10.42 (5.80,18.72)	1 9.57 (4.91,17.20)***
Decider on type of contraceptive to be used				
Self/husband Both wife and husband jointly	182 357	49 191	2.00 (1.39,2.87) 1	1.59 (0.95,2.39) 1

^a^ adjusted for age, education, occupation, regular media exposure, number of pregnancy, decider on number of children to have, knowledge on LAPMs, attitude towards LAPMs use, ever use of modern contraception methods, husband’s attitude towards LAPMs use and decider on type of contraceptive to be used

*Significant at *P* < 0.05, **significant at *P* < 0.01, ***significant at *P* < 0.001

However, the merchant category of the women’s occupation variable was negatively associated with desire for birth spacing or limiting. That is, women who were merchants in occupation (AOR = 0.52, 95 % CI: [0.34, 0.79]) were less likely to desire for birth spacing or limiting compared to housewives (Table 4).

Of the several factors included in the multivariate logistic regression analysis to identify factors associated with having desire for birth spacing or limiting but not using LAPMs, women’s education, husband’s attitude towards LAPMs, discussion on FP with husband, knowledge, attitude and intention to use LAPMs were positively associated with not using LAPM among women with desire for birth spacing or limiting. That is, women’s education; secondary education (AOR = 3.05, 95 % CI: [1.18, 7.85]), not discussing family planning methods with husband (AOR = 5.93, 95 % CI: [3.61, 8.77]), low knowledge (AOR = 2.51, 95 % CI: [1.08, 5.85]), negative attitude towards LAPMs (AOR = 2.00, 95 % CI: [1.02, 3.87]), unfavorable/unknown husband’s attitude towards LAPMs use (AOR = 3.60, 95 % CI: [3.06, 5.84]) and not intending to use LAPMs (AOR = 7.70, 95 % CI: [4.18, 14.17]) were more likely not using LAPMs compared to their counterparts. However, women’s occupation; daily labor (AOR = 0.31, 95 % CI: [0.12, 0.82]) was negatively associated with LAPMs use (Table 5).

**Table 5:** Factors associated with not using long acting and permanent contraceptive methods among married women of reproductive age who desire birth spacing or limiting in Aksum town, North Ethiopia, 2015, (*N* = 539)

Variables	LAPMs use		COR (95 % CI)	AOR (95 % CI)^a^
	No (n)	Yes (n)		
Age				
< 25 25-34 ≥ 35	86 201 172	24 33 23	1 1.70 (0.95, 3.05) 2.08 (1.11, 3.89)	1 0.75 (0.35, 1.60) 1.24 (0.64, 2.40)
Educational status				
No formal education Primary education Secondary education Above secondary education	116 170 131 42	14 33 19 14	2.74 (1.21, 6.22) 1.72 (0.84, 3.50) 2.30 (1.06, 4.98) 1	2.30 (0.75, 7.04) 1.87 (0.73, 4.84) 3.05 (1.18, 7.85)* 1
Occupation				
Daily laborer Merchant Employed Student Housewife	22 65 57 7 308	9 15 11 1 44	0.35 (0.15, 0.81) 0.62 (0.33, 1.18) 0.74 (0.36, 1.52) 1.00 (0.12, 8.32) 1	0.31 (0.12, 0.82)* 0.69 (0.33, 1.44) 1.47 (0.59, 3.68) 1.38 (0.12, 15.55) 1
Decider on number of children to bear				
Self/husband Both wife & husband jointly	89 370	7 73	1 2.5 (1.12, 5.59)	1 1.54 (0.93, 2.48)
Discussed FP methods with husband				
Yes No	304 155	74 6	1 6.25 (2.66, 14.69)	1 5.93 (3.61, 8.77)***
Knowledge on LAPMs				
High	173	41	1	1
Medium	124	26	1.13 (0.66, 1.95)	1.44 (0.76, 2.72)
Low	162	13	2.95 (1.53, 5.71)	2.51 (1.08, 5.85)*
Attitude towards LAPMs				
Positive	224	60	1	1
Negative	235	20	3.15 (1.84, 5.39)	2.00 (1.02, 3.87)*
Husband’s attitude to LAPMs use				
Approve Disapprove/don’t know	275 184	63 17	1 2.48 (1.41, 4.37)	1 3.60 (3.06, 5.84)***
Decider on type of contraceptive to be used				
Self/husband Both wife & husband jointly	172 287	10 70	4.20 (2.12, 8.42) 1	2.58 (0.96, 4.20) 1
Intention to use LAPMs				
Yes No	156 303	63 17	1 7.20 (4.07, 12.72)	1 7.70 (4.18, 14.17)***

^a^ adjusted for age, education, occupation, decider on number of children to have, discussed FP methods with husband, Knowledge on LAPMs, husband’s attitude to LAPMs use, decider on type of contraceptive to be used and Intention to use LAPMs

*Significant at *P* < 0.05, **significant at *P* < 0.01, ***significant at *P* < 0.001

## Discussion

In this study total desire for birth spacing or limiting was 69.2 % (95 % CI: 65.9, 72.4) which is higher than the total desire in developing region (57 %) [5]. However, it is lower than the 2011 EDHS report (75 %) [10] and a study in Ethiopia (77.8 %) [21]. Of those who desire to space or limit births, 85.2 % were not using LAPMs which is higher than the overall non-use of LAPMs in developing region (53 %) [5] and study in Ethiopia (62.9 %) [13]. The inconsistency could be due to difference in time, socio-cultural and access to information and the services.

Age and number of lifetime pregnancy were positively associated with desire for birth spacing or limiting. Increasing age and number of lifetime pregnancy might let women have many children and met their fertility desire which results in desire for birth spacing or limiting and use LAPMs [13, 16, 17, 19, 20, 26]. Women’s education was positively associated with both desire for birth spacing or limiting and use of LAPMs as shown in studies elsewhere [17, 18, 21, 24, 28, 29, 32]. This could be due to difference in awareness and health seeking behavior. Moreover, desire for birth spacing or limiting and not using LAPMs also varied with occupation [17, 18]. This might be due to difference in socioeconomic status, access to information and women empowerment [17, 26, 33].

Women with regular media exposure had higher desire for birth spacing or limiting compared to their counterparts similar to studies in Ethiopia [22]. Moreover, non-use of LAPMs varied with women’s knowledge and attitude. Women with low knowledge on LAPMs were more likely not using LAPMs than their counterparts as shown in studies done in Ethiopia and Rwanda [15, 27]. Likewise, women who had negative attitude towards LAPMs were more likely not using LAPMs which was similar to studies in Ethiopia and Rwanda [17, 27]. This could be due to difference in health seeking behavior and acceptance of LAPMs.

Women who decide jointly with their husband on the number of children to bear were more likely to desire for birth spacing or limiting than their counterparts. Moreover, women who had no discussions about FP methods with their husbands were more likely not using LAPMs which is similar to studies elsewhere [12, 17, 18, 21, 27, 28, 34]. This could be due contribution of men’s involvement and exchange of ideas on FP which leads to desire for birth spacing or limiting and LAPMs use [12, 16]. Furthermore, women having husbands with unfavorable/unknown attitude towards use of LAPMs were more likely to desire for birth spacing or limiting compared to their counterparts. This is consistent with studies in Ethiopia and Rwanda [13, 27]. Similar pattern of relationship was found between husband’s attitude towards LAPMs and not using LAPMs as shown in studies elsewhere [12, 16, 17, 21, 27]. Having husband with unfavorable attitude towards LAPMs let women lack their husband’s support in family planning and have too many or too close births, which results in higher desire for birth spacing or limiting.

This study has its own drawbacks. The study suffers from the usual limitation of cross sectional study. Moreover, it missed qualitative data and did not assess the contribution of health service related factors. It is still not free from social desirability and recall biases.

## Conclusion

This study revealed that there was high desire for birth spacing or limiting and not using LAPMs among women who had desire for birth spacing or limiting. Education, occupation, husband’s attitude towards LAPMs, age, number of pregnancy, regular media exposure, and decider on the number of children to bear were significantly associated with desire for birth spacing or limiting. Moreover; education, occupation, husband’s attitude towards LAPMs, discussion on FP with husband, knowledge, attitude and intention to use LAPMs were significantly associated with not using LAPM. In conclusion, findings from this study suggested the need to educate mothers on LAPMs with emphasis on those with higher number of children and lower educational status. The findings of this study also underlined the need for male involvement in FP program. Thus, the federal ministry of health and regional health bureau in combination with NGOs working on FP has to work hard to increase accessibility and availability of LAPMs in the study area.

## Abbreviations

AOR: Adjusted odds ratio
CI: Confidence interval
COR: Crude odds ratio
EDHS: Ethiopian Demographic and Health Survey
ETB: Ethiopian birr
FP: Family planning
IUCDs: Intrauterine contraceptive devices
LAPMs: Long acting and permanent contraceptive methods
NGOs: Nongovernmental Organizations
SD: Standard deviation
